# Geographic Disparity in Asthma Hospitalizations: The Role of Race/Ethnicity, Socioeconomic Status, and Other Factors

**DOI:** 10.7759/cureus.20015

**Published:** 2021-11-29

**Authors:** Noah De La Cruz, Jonathon H Hines, Chip Shaw, Duke Appiah

**Affiliations:** 1 College of Osteopathic Medicine, Sam Houston State University, Conroe, USA; 2 Public Health, Texas Tech University Health Sciences Center, Lubbock, USA

**Keywords:** socioeconomic status, particulate matter, race/ethnicity, epidemiology, hospitalizations, asthma

## Abstract

Background

In the United States, asthma is the most common chronic disease in children, and is associated with low sociodemographic, economic, and environmental factors.

Objective

To investigate geographic disparities in asthma hospitalizations and the roles that race/ethnicity, health insurance, and other environmental factors played on these disparities in Lubbock County, Texas.

Methods

Data were obtained from the Texas Inpatient Public Use Data File for the years 1999-2018. International classification of disease codes were used to identify primary diagnoses of asthma among all severe inpatient admissions. Logistic regression was used to estimate odds ratios (ORs) and 95% confidence intervals (CIs).

Results

Of the 248,768 patients admitted for severe conditions, 4,224 had a primary diagnosis of asthma. In multivariable-adjusted models, the odds of asthma hospitalizations varied across geographic regions of Lubbock with the Northeast having the highest age-adjusted prevalence (7.17 per 1,000) and ORs for asthma hospitalizations (OR: 1.25, CI: 1.12-1.40). Data suggested that non-Hispanic Blacks using federal insurance in the Northeast region had the highest odds for asthma hospitalizations (OR: 4.88, CI: 3.06-7.79; p-interaction = 0.001). Across all regions, a 1 μg/m^3^ increase in particulate matter 2.5 was associated with a 27% higher likelihood of asthma hospitalization (OR: 1.27, 95% CI: 1.23-1.31).

Conclusion

In this study, geographic disparities in asthma hospitalizations were observed within Lubbock County and were significantly influenced by a disparate distribution of socioeconomic factors related to health insurance and race/ethnicity. The potential contributory role of particulate matter needs further investigation.

## Introduction

Asthma, an incurable chronic disease involving the inflammation of the bronchial tubes, is a major and ever-growing public health issue that most often results in decreased quality of life, worldwide [1]. In the United States, the occurrence of asthma is higher among children (9.5%) than adults (7.7%) regardless of age, sex, and ethnicity, making it the most common chronic disease among children [2]. Asthma accounts for substantial absenteeism from school than any other chronic disease in children [3]. The prevalence of asthma is increasing nationwide. From 2001 to 2017, the prevalence of asthma increased by nearly 8% [4]. Asthma also poses a large financial burden on those afflicted. In 2013, the total estimated cost of asthma due to missed work and school days, as well as mortality, was estimated to be more than $81.9 billion [5].

Although the incidence of asthma in the United States has been increasing across all ages, sexes, and races, disparities in asthma hospitalizations and mortality by race/ethnicity have been reported [2]. Compared to all other races, non-Hispanic Blacks disproportionately bear a greater burden of asthma. Furthermore, in 2019, non-Hispanic Black children were estimated to have a mortality rate that is eight times higher than non-Hispanic White children [6]. The burden of asthma is also high among the Hispanic population. Recent reports on the prevalence of asthma among children show that 13.5% and 7.5% of non-Hispanic Black and Hispanic children, respectively, currently have asthma compared to 6.4% of non-Hispanic White children [7].

Previous studies have shown an association between prolonged exposure to low-socioeconomic environments with a higher prevalence of asthma as well as exposure to particulate matter (PM 2.5) and risk of asthma exacerbation [8-10]. In the United States, approximately 45% of non-Hispanic Black poor children and 35% of Hispanic poor children reside in areas of low socioeconomic status, which is more than double the estimated percentage for non-Hispanic White poor children (12%) [11]. Low socioeconomic status and increased exposure to air pollution may allow for a greater risk of asthma as well as asthma hospitalization in children [12,13]. With an increasing burden of asthma over the years, it has become increasingly important to analyze the prevalence of asthma, and how it relates to geographic regions in order to better understand the socioeconomic and environmental risk factors that contribute to asthma hospitalization.

Therefore, this study aimed to investigate geographic disparities in asthma hospitalizations and evaluate the roles that race/ethnicity, health insurance, and other environmental factors played on these disparities in Lubbock County, a county in west Texas which is a large medically underserved area [14].

## Materials and methods

Data were obtained from the Texas Health and Human Services (THHS) Inpatient Public Use Data File for the years 1999-2018. This database contains discharge information on all patients admitted to a hospital in Texas. Admissions were classified as emergency, urgent, elective, newborn, trauma, or information not available. All emergency admissions were considered to be severe admissions. For this analysis, information was limited to residents of Lubbock County, which is an urban county of approximately 310,000 residents with 53% of them being non-Hispanic Whites, 36% being Hispanics, 8% being non-Hispanic Blacks, and 3% being of Asian, Native Hawaiian, or Pacific Islander race and ethnicity [15]. Lubbock County resides in west Texas, which is largely a medically underserved area [14]. Because the data were both de-identified and publicly available, ethical approval from an institutional review committee was not required. 253,110 patients aged 1-89 had admissions that were considered severe. From these patients, we excluded those with missing information for race (n=88) or those who reported a Zoning Improvement Plan (zip) code (79408) designated for only postal office boxes (n=4,254), which resulted in an analytic sample of 248,768 patients.

**Figure 1:**
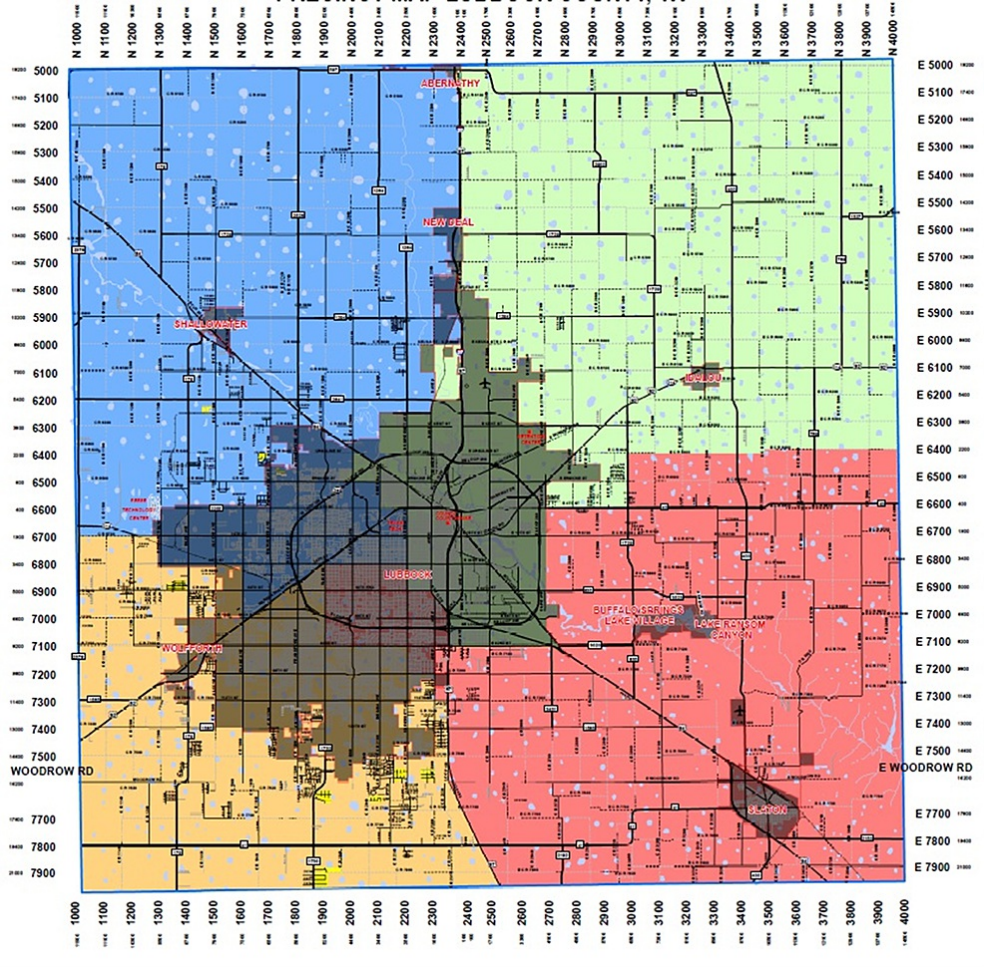
Geographic regions of Lubbock County. The geographic regions of Lubbock County were defined by zip codes to match the major precincts of the county. Map adopted from the official website of Lubbock County, Texas which is available at https://www.co.lubbock.tx.us/department/division.php?structureid=179.

International Classification of Diseases ninth and tenth revision codes were used to identify cases with a primary diagnosis of asthma (ICD-9-CM: 49300, 49301, 49302, 49310, 49311, 49312, 49320, 49321, 49382, 49390, 49391, 49392; ICD-10-CM: J45, J452, J4520, J4521, J4522, J453, J4530, J4531, J4532, J454, J4540, J4541, J4542, J455, J4550, J4551, J4552, J459, J4590, J45901, J45902, J45909, J4599, J45990, J45998) and other comorbid conditions. Zip codes were used to classify region of residence in Lubbock County into Northwest, Northeast, Southwest, and Southeast regions (Figure 1). The geographic regions of Lubbock County were defined by zip codes to match the major precincts of the county [16].

Demographic and behavior lifestyle factors obtained included age (classified into the following groups: 1-17 years, 18-29 years, 30-64 years, and ≥ 65 years), sex, race, insurance, obesity, and tobacco use. All these variables were obtained from the THHS Inpatient Public Use Data File. Health insurance status was used as a proxy for socioeconomic status. Some studies have shown insurance status to be a good proxy for socioeconomic status [17]. Federal insurance was defined as Medicare and Medicaid. These insurance options are both provided by the United States government for people that meet the necessary criteria. Medicare is a federal insurance option for those older than 65 years of age or under 65 with a qualifying disability. Medicare is split into parts A, B, and D. Medicare Part A is considered hospital insurance. Medicaid is an assistance program that helps low-income people of all ages and is run by individual state governments under federal guidelines. Some patients may be considered dual eligible and receive assistance via both Medicare and Medicaid [18]. Children’s Health Insurance Program is another assistance program specifically for children that is operated by state governments under federal requirements [19]. PM 2.5 levels were obtained for Lubbock County from the National Environmental Public Health Tracking Network for the years of 2001-2014 [20].

Chi-square tests were used to compare the characteristics of age, sex, race/ethnicity, region of residence, and insurance status between patients who experienced a severe asthma hospitalization to patients who experienced a severe hospitalization for all other non-asthma-related conditions. Crude and age-adjusted prevalence of severe asthma hospitalizations were calculated for the various regions of Lubbock County using the 2010 US census populations.

 Logistic regression was used to estimate odds ratios (ORs) and 95% confidence intervals (CIs) for the association between region of residence and severe asthma hospitalization. To account for the possibility of individual characteristics confounding any observed associations, the logistic regression models were adjusted for age, sex, race, health insurance status, smoking status, upper respiratory infection, allergic rhinitis, eczema, and obesity. Because certain characteristics within regions may influence geographic disparities in asthma hospitalizations, we tested for interactions between race/ethnicity or socioeconomic status with geographic regions. Only significant interactions (p < 0.05) are reported.

Sensitivity analyses were conducted to explore the relationship of fine particulate matter with all asthma hospitalizations in Lubbock County to provide some context for the findings from this study relating geographic region to severe asthma hospitalizations using generalized estimation equations. A two-tailed probability value less than 0.05 was considered statistically significant. All statistical analyses were performed using SPSS v.24.0 (IBM Corp., Armonk, NY, USA).

## Results

Of the 248,768 patients admitted for severe conditions, 4,224 had a primary diagnosis of asthma. Approximately 52% of patients who experienced a severe asthma hospitalization were in the age group of 1-17 years, with 54.5% of patients being males. The racial/ethnic distribution of patients was as follows: 36.5% were non-Hispanic White, 25.1% were non-Hispanic Black, 33.7% were Hispanic, and 4.7% were of other races or ethnicities (Table 1). The crude prevalence of severe asthma hospitalizations (per 1,000 individuals) by region was as follows: Northwest: 1.68, Northeast: 2.52, Southwest: 1.54, Southeast: 2.03 (Table 2). These prevalence estimates increased when age-standardized to the 2010 population of Texas. Thus, the age-adjusted prevalence of severe asthma hospitalizations per 1,000 individuals was 4.38 for the Northwest, 7.17 for the Northeast, 4.41 for the Southwest, and 5.55 for the Southeast regions (Table 2).

**Table 1:** Characteristics of 248,768 emergency hospitalizations, Texas Inpatient Use Data File 1999-2018.

	Primary Diagnosis		
	Severe Asthma	No Asthma	p-Value
	(n = 4,224)	(n = 244,544)	
Age, %			<0.001
1-17	51.8	5.2	
18-29	11.0	8.2	
30-64	30.4	42.5	
≥65	6.8	44.1	
Male, Sex, %	54.5	54.3	<0.001
Race/Ethnicity, %			<0.001
Non-Hispanic White	36.5	59.1	
Non-Hispanic Black	25.1	9.9	
Hispanic	33.7	25.2	
Other	4.7	5.8	
Region, %			<0.001
Northwest	20.6	21.2	
Northeast	16.0	10.9	
Southwest	51.9	58.1	
Southeast	11.5	9.8	
Primary Payment Source, %			<0.001
Commercial	9.1	9.4	
Federal	32.8	42.8	
Private	17.3	15.2	
Self-Pay	11.8	9.2	
Other	29.0	23.4	

**Table 2:** Prevalence (per 1,000) of primary diagnosis of severe asthma by region. *The denominator is the total number of emergency hospital admissions in each region. ^†^The denominator is the regional population from 2010 census. ^‡^The denominator is the Texas population from 2010 census.

Region	Crude Rate*	Crude Rate^†^	Age-Adjusted Rate^‡^
Northwest	1.68	1.36	4.38
Northeast	2.52	3.35	7.17
Southwest	1.54	1.37	4.41
Southeast	2.03	2.36	5.55

Table 3 describes the characteristics of patients hospitalized for severe exacerbations of asthma by region in Lubbock County. Compared to patients from the other three regions, a greater proportion of individuals from the Northeast region were non-Hispanic Black (56.2%), with a greater proportion of them being less than 18 years of age (58.9%), and used federal insurance (43.3%) (Table 3). A statistically significant association was found between geographic region of residence and severe asthma hospitalization (p < 0.001). In unadjusted analyses, residents of the Northeast region had 50% higher odds (OR: 1.50, 95% CI: 1.35-1.66) of having severe asthma hospitalization, compared to residents from the Northwest region. Residents of the Southeast region also had 21% higher odds (OR: 1.21, 95% CI: 1.08-1.35) for severe asthma hospitalizations (Table 4). However, after adjustment for age, sex, race and ethnicity, socioeconomic status, smoking, upper respiratory infection, allergic rhinitis, eczema, and obesity, the association between region of residence and severe asthma hospitalizations was limited to only the region of Northeast (p = 0.001). Residents of the Northeast region had a 25% higher odds of severe asthma hospitalization (OR: 1.25, 95% CI: 1.12-1.40) (Table 4).

**Table 3:** Characteristics of severe asthma hospitalizations of Lubbock County by region, Texas Inpatient Use Data File 1999-2018.

			Regions		
Characteristics	Northwest	Northeast	Southwest	Southeast	Overall
	(n = 869)	(n = 676)	(n = 2,193)	(n = 486)	(n = 4,224)
Age, %					
1-17	49.25	58.88	49.89	54.44	51.8
18-29	14.04	8.14	11.22	8.67	11.0
30-64	31.07	27.96	30.96	29.23	30.4
≥65	5.64	5.03	7.93	5.65	6.8
Male, Sex %	49.02	43.79	45.23	41.53	54.5
Race, %					
Non-Hispanic White	36.9	15.7	46.8	17.5	36.5
Non-Hispanic Black	10.8	56.2	19.1	33.9	25.1
Hispanic	47.3	22.6	30.1	40.5	33.7
Other	4.9	5.5	4.0	6.1	4.7
Primary Payment Source, %					
Commercial	9.21	7.39	9.94	7.26	9.1
Federal	28.42	43.34	30.46	35.69	32.8
Medicare	25.1	14.7	23.1	26.0	22.0
Medicaid	74.1	85.3	75.0	74.0	76.9
Private	19.56	11.83	19.74	9.27	17.3
Self-Pay	13.81	12.57	10.62	12.50	11.8
Other	28.99	24.85	29.23	33.27	29.0
Other Respiratory Disease, %					
Upper Respiratory Infection	7.02	7.25	7.79	7.41	7.50
Allergic Rhinitis	2.07	0.89	1.59	1.65	1.59
Eczema, %	0.46	0.74	0.27	0.41	0.40
Tobacco Use, %	12.43	10.06	12.45	11.73	11.98

**Table 4:** ORs and 95% CIs for the association of region with primary severe asthma diagnosis, 1999-2018, Lubbock County, TX. OR, odds ratio; CI, confidence interval. N: number (and %) of primary asthma diagnosis by region. Model 1: unadjusted. Model 2: adjusted for age, sex, and race/ethnicity. Model 3: adjusted for age, sex, race/ethnicity, insurance, and smoking status. Model 4: adjusted for age, sex, race/ethnicity, insurance, smoking status, upper respiratory infection, allergic rhinitis, eczema, and obesity.

		Model 1	Model 2	Model 3	Model 4
Region	N (%)	OR (95% CI)	OR (95% CI)	OR (95% CI)	OR (95% CI)
Northwest	869 (20.6)	1	1	1	1
Northeast	676 (16.0)	1.50 (1.35-1.66)	1.26 (1.13-1.40)	1.25 (1.12-1.40)	1.25 (1.12-1.40)
Southeast	486 (11.5)	1.21 (1.08-1.35)	1.10 (0.98-1.23)	1.09 (0.97-1.23)	1.10 (0.98-1.24)
Southwest	2,193 (51.9)	0.92 (0.85-0.99)	1.04 (0.96-1.13)	1.05 (0.96-1.14)	1.04 (0.96-1.13)
p-Value		0.001	0.001	0.001	0.001

**Figure 2:**
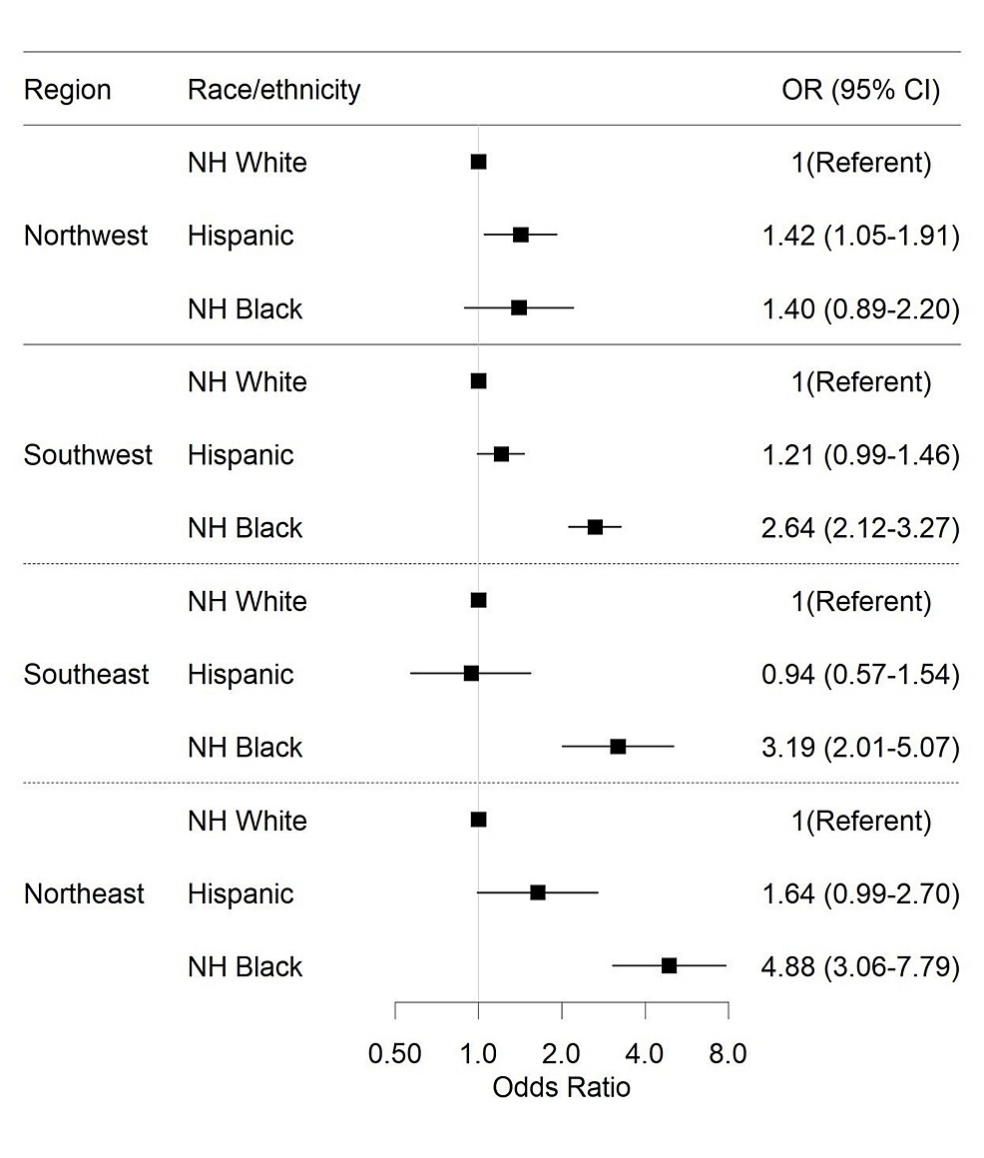
The relation of geographic location, race/ethnicity, and health insurance status with severe asthma hospitalizations in Lubbock County, 1999-2018. NH, non-Hispanic; OR, odds ratio; CI, confidence interval.

Overall, the OR of asthma hospitalization was higher among racial/ethnic minority groups with Hispanics (OR = 1.09; CI: 1.01-1.18) and non-Hispanic Blacks (OR = 2.43; CI: 2.22-2.66) having elevated odds compared to non-Hispanic Whites. There was a significant interaction between region and race (p = 0.001) as well as region and socioeconomic status as assessed by insurance status (p = 0.001). Comparing non-Hispanic Blacks to Hispanics, the OR for asthma hospitalization was highest in the Northeast region (OR = 2.98; CI: 2.44-3.64), followed by the Southwest (OR = 2.46; CI: 2.15-2.81), Southeast (OR = 2.14; CI: 1.72-2.67), and Northwest (OR = 1.24; CI: 0.98-1.57) regions. Among persons with federal health insurance, compared to the Northwest region, the OR for asthma hospitalization was again highest among persons from the Northeast (OR = 1.52; CI: 1.26-1.82) with no significant estimates reported for the Southeast (OR = 1.17; CI: 0.95-1.43) and Southwest (OR = 1.10; CI: 0.94-1.28) regions. The joint association of race/ethnicity and insurance status varied by region (p for three-way interaction = 0.001) with the association observed to be highest in the Northeast region (Figure 2).

Finally, in sensitivity analyses, we observed that a 1 μg/m^3^ increase in PM 2.5 was associated with a 27% higher odds of asthma hospitalization (OR: 1.27, 95% CI: 1.23-1.31).

## Discussion

In this study, geographic region of residence in Lubbock County was associated with severe asthma hospitalization with patients from the Northeast region having higher odds for severe asthma hospitalization. Other factors such as race/ethnicity and socioeconomic status as determined by health insurance status were observed as possible modifiers of the association of geographic region with severe asthma hospitalizations.

Asthma presents a growing problem as the burden of cost attributed to it also continues to increase. Currently, about 7.0% of children (479,712) in Texas have current asthma, and nearly $89.5 million were spent through Medicaid reimbursement among beneficiaries with asthma [21]. Therefore, identifying and understanding contributing factors that might be related to the spatial distribution of asthma prevalence and its complications are very important to the economies of many counties. In the current study, geographic location was observed to be positively associated with asthma hospitalizations; it was observed that residents of the Northeast region had higher odds of being hospitalized with severe asthma, and this was partly due to a greater proportion of residents of this region being of low socioeconomic status than the other three regions of Lubbock County [15]. For this study, health insurance status was used as a surrogate for socioeconomic status. Previous studies have shown insurance status to be an effective surrogate for other social determinants of health such as socioeconomic status and are more effective in determining an individual’s long-term health status due to most social factors not being routinely included in the clinical setting [17].

It is widely known that racial and ethnic disparities in health insurance coverage account for a sizable difference in access to care [22]. Previous studies have shown that non-Hispanic Black and Hispanic populations are more likely to utilize public or federal insurance coverage and are less likely to have private insurance [23,24]. The use of federal insurance programs such as Medicare creates economic challenges due to their inability to cover or greatly reduce the costs of prescription drugs that are of great importance in the management of asthma [25]. Since chronic exposure to low-socioeconomic environments is associated with the development of asthma and persistent asthma episodes, this characteristic is likely a contributing factor as to why the highest burden of severe asthma hospitalizations was highest in the Northeast region of Lubbock that had a greater proportion of residents being of racial/ethnic minority groups [8].

Geographic disparities in asthma hospitalizations are unlikely to be affected solely by race/ethnicity and health insurance status. Other potential factors such as air pollution can also contribute to asthma hospitalizations. Some studies have shown how residential dwellings in areas with more manufacturing sites are associated with an increased risk of acute respiratory distress syndrome due to their ability to produce airborne toxins which affect the respiratory health of nearby residents [26,27]. Other studies have also shown that non-Hispanic Black populations experience a greater risk of hazardous air pollutants such as those that come from traffic sources due to residential segregation that has forced living in areas of greater exposure to air pollution [13,28]. In Lubbock, there is a higher concentration of industrial activity, heavy manufacturing, and environmental hazards in neighborhoods located in the Northeast region. A previous study observed that census blocks with a high-density African American population were located at an average of 568.1 meters closer to toxic-releasing facilities than the average block groups in Lubbock County [29].

With air pollution being an important environmental factor that contributes to asthma hospitalizations [30], the relation of PM 2.5 levels with asthma hospitalizations was estimated with data obtained for Lubbock County from the National Environmental Public Health Tracking Network for the years of 2001-2014. It was observed that a one-unit increase in PM 2.5 was associated with 27% higher odds of having an asthma diagnosis. Although data on regional variation in particulate matter were not available, it was worth noting that the Northeast region of Lubbock has concrete batch plants that discharge particles created by the crushing of stone. A study conducted in Lubbock County identified spatial environmental inequality between geographic regions in Lubbock [29]. Therefore, we speculate that another possible reason for the high occurrence of asthma in the Northeast region could be due to air pollution with residents of this region possibly being exposed to higher levels of PM 2.5 than all other regions. Further studies are warranted to understand how differences in particulate matter across the various regions of Lubbock County translate to health outcomes. Such information will be critical for the design of interventions to reduce the regional, racial/ethnic, and socioeconomic discrepancies in asthma hospitalizations in various communities within and outside of Lubbock County.

This study has many strengths with the most notable being the use of a large statewide database with information that is representative of inpatient data for all hospitals in Lubbock County for the years of 1999-2018. The data used in this study also had information on primary and other diagnoses of pre-existing conditions. This provided the opportunity to control for several factors that could potentially confound the associations reported.

This study also has limitations, which include the following: data on exposure to secondhand smoke were not available, so its influence on asthma occurrence could not be determined in this study. The rate of domicile relocation for Lubbock County was also not available in the database. Therefore, it is unknown if participants were likely to stay in the same zip code or moved to different zip codes during the period of this study, thus being exposed to different levels of PM 2.5 during the years of study. However, with a limited number of healthcare facilities and occupations outside of Lubbock County, the possibility of a large number of residents of Lubbock County relocating outside of the county is likely minimal. Although an association of PM 2.5 and asthma was found for the entirety of Lubbock County, PM 2.5 levels were unavailable for the various geographic regions in the county. The data used for this study did not contain individualized information on hospitalizations; therefore, the frequency of visits by a single patient could not be evaluated.

The findings of this study have important clinical and public health significance. Currently, most efforts to reduce asthma hospitalizations have focused on management of the disease. The results of the current study highlight the need for intervention programs that consider how neighborhood-level factors may be critical in reducing the burden of asthma hospitalizations.

## Conclusions

In conclusion, geographic disparities in asthma hospitalizations in Lubbock County were observed in this study, which was significantly influenced by a disparate distribution of socioeconomic factors related to health insurance and race/ethnicity. This study also found that individuals who were non-Hispanic Black were more likely to have hospitalizations for asthma. Other contributing environmental factors such as particulate matter observed to be associated with severe asthma hospitalization require further investigation for confirmation.
